# Radiosensitization with combined use of olaparib and PI-103 in triple-negative breast cancer

**DOI:** 10.1186/s12885-015-1090-7

**Published:** 2015-03-03

**Authors:** Na Young Jang, Dan Hyo Kim, Bong Jun Cho, Eun Jung Choi, Jong-Soo Lee, Hong-Gyun Wu, Eui Kyu Chie, In Ah Kim

**Affiliations:** 1 Department of Radiation Oncology, Graduate School of Medicine, Seoul National University, Seoul, Korea; 2 Medical Science Research Institute, Seoul National University Bundang Hospital, Seongnam, Korea; 3 Department of Life Science, College of Natural Sciences, Ajou University, Suwon, Korea; 4 Department of Radiation Oncology, Seoul National University, Seoul, Korea; 5 Cancer Research Institute, Seoul National University, Seoul, Korea; 6 Department of Radiation Oncology, Veterans Health Service Medical Center, Seoul, Korea

**Keywords:** Triple-negative breast cancer, Radiotherapy, Olaparib, PI-103, PARP inhibitor, PI3K inhibitor

## Abstract

**Background:**

Triple-negative breast cancer (TNBC) shows aggressive clinical behavior, but the treatment options are limited due to lack of a specific target. TNBC shares many clinical and pathological similarities with BRCA-deficient breast cancer, for which poly(ADP-ribose) polymerase (PARP) inhibitor is effective, but PARP inhibitor alone failed to show clinical effects in patients with sporadic TNBC. Radiation induces DNA double-strand breaks, and the phosphoinositide 3-kinase (PI3K) signaling pathway has been known to regulate steady-state levels of homologous recombination. A recent preclinical study showed that PI3K inhibition impairs BRCA1/2 expression and sensitizes BRCA-proficient TNBC to PARP inhibition. Therefore, we assessed the radiosensitizing effect, and the underlying mechanism of combination treatment with PARP inhibitor olaparib and PI3K inhibitor PI-103 in BRCA-proficient TNBC cells.

**Methods:**

MDA-MB-435S cells were divided into four treatment groups, irradiation (IR) alone, olaparib plus IR, PI-103 plus IR, and olaparib plus PI-103 plus IR. Cells were exposed to the drugs for 2 hours prior to irradiation, and the cell survival curve was obtained using a clonogenic assay. Western blotting and immunofluorescent detection of γH2AX foci were performed. Xenograft and bioluminescence imaging were carried out to assess in vivo radiosensitivity.

**Results:**

Combined use of olaparib and PI-103 enhanced radiation-induced death of MDA-MB-435S (sensitizer enhancement ratio[SER]_0.05_,1.7) and MDA-MB-231-BR (SER_0.05_,2.1) cells and significantly reduced tumor volume in a xenograft models (*P* < 0.001). Treatment with PI-103 showed persistent γH2AX foci, indicating delayed repair of DNA strand breaks. PI-103 alone increased levels of poly(ADP-ribose) and phosphorylated extracellular signal-regulated kinase, and downregulated BRCA1.

**Conclusions:**

Combined use of olaparib and PI-103 enhanced radiation-induced cell death in BRCA-proficient MDA-MB-435S and MDA-MB-231-BR cells and xenografts. TNBC patients have high incidences of locoregional relapse and distant metastasis, and radiation therapy targets both locoregional control and treatment of distant recurrences such as brain metastasis or other oligometastasis. Targeting of the PI3K signaling pathway combined with PARP inhibition maybe a feasible approach to enhance effects of radiation in BRCA-proficient TNBC.

## Background

Triple-negative breast cancer (TNBC) is defined as a tumor that does not express the estrogen receptor (ER), progesterone receptor, or human epidermal growth factor receptor 2 (HER2). Aggressive clinical behaviors, such as early distant metastasis and lack of specific treatment targets, i.e. ER or HER2, have been obstacles to the treatment of TNBC [1]. Cytotoxic chemotherapy or combination with a targeted agent, such as bevacizumab or cetuximab, has been used, but the results were disappointing [2]. Meanwhile, the introduction of poly(ADP-ribose) polymerase (PARP) inhibitors were expected to provide a promising new therapeutic strategy for TNBC.

The PARP family of enzymes is involved in DNA repair, cell proliferation and death, and genomic stability [3-5]. PARP1 is the most abundant PARP and is involved in base-excision repair (BER) [6]. When a DNA strand break occurs, PARP1 rapidly binds to the break site and induces auto-poly(ADP-ribosyl)ation. Auto-poly(ADP-ribosyl)ation creates a negative charge, which recruits the enzymes required for BER [6,7]. Synthetic lethality occurs when two otherwise nonlethal mutations together result in an inviable cell [8]. When DNA damage occurs, cells with mutations in either PARP or BRCA, which is involved in homologous recombination (HR), can survive, whereas cells with mutations in both cannot [9].

This strategy is very effective for treatment of breast cancer patients with BRCA mutations, but the problem is that incidence of BRCA-related breast cancer is less than 10% [10]. Recently, some investigators have used the term “BRCA-ness” of TNBC, because BRCA-deficient breast cancer and sporadic basal-like or TNBC share many clinical and pathological similarities [11,12]. Exploiting these similarities, clinical trials have tested the effectiveness of PARP inhibitors on BRCA-proficient TNBC patients. However, PARP inhibitor alone failed to show clinical effects in patients with TNBC [13]. Subsequent studies are ongoing to test the efficacy of the combined use of PARP inhibitor and cytotoxic chemotherapy or radiotherapy.

Radiation induces DNA double-strand breaks (DSBs), and the phosphoinositide 3-kinase (PI3K) signaling pathway has been known to regulate steady-state levels of HR [14]. Furthermore, Ibrahim et al. published interesting research results showing that PI3K inhibition impairs BRCA1/2 expression and sensitizes BRCA-proficient TNBC to PARP inhibition [15]. Inhibition of the PI3K signaling pathway induces feedback upregulation of extracellular signal-regulated kinase (ERK) and subsequent increased activation of ETS1 as an ERK-related transcription factor. ETS1 suppresses BRCA1/2 expression and impairs HR, thereby sensitizing the cells to the PARP inhibitor. In addition, preclinical studies showed increased radiosensitivity with use of the PARP inhibitor in replicating cells [16].

Taken together, these findings show that radiation, PI3K inhibitors, and PARP inhibitors may enhance each other’s tumor cell killing effects, and we postulated that the combined use of a PARP inhibitor and PI3K inhibitor would sensitize cells to radiation. Therefore, we assessed the radiosensitizing effect of combined treatment of BRCA-proficient TNBC cells with olaparib and PI-103and investigated the underlying mechanism of action.

## Methods

### Cell culture

Triple-negative, BRCA-proficient breast cancer cell lines MDA-MB-435S and MDA-MB-231-BR (American Type Culture Collection, Rockville, MD, USA) were cultured in RPMI 1640 medium (Gibco; Invitrogen, Carlsbad, CA, USA) supplemented with 10% fetal bovine serum (Gibco; Invitrogen) at 37°C in an atmosphere of 95% air and 5% CO_2_.

### Pharmacologic inhibitors

Olaparib was obtained from Selleck Chemicals (Houston, TX, USA) and PI-103 (pyridinylfuranopyrimidine inhibitor) was obtained from Calbiochem (Billerica, MA, USA). Drugs were diluted in dimethylsulfoxide (DMSO).

### Short interfering RNA (siRNA) transfection

BRCA1 siRNA was obtained from BioNeer (Alameda, CA, USA). Cells were plated in six-well plates and transfected with 100 nM BRCA1 siRNA using Lipofectamine® RNAiMAX transfection reagent (Life Technologies, Grand Island, NY, USA) according to the manufacturer’s protocol.

### Clonogenic assay

The clonogenic assay was carried out according to a previously described protocol [17]. Appropriate numbers of cells were plated across the different treatment groups for each radiation dose. Treatment groups were as follows; irradiation (IR) alone, olaparib and IR, PI-103 and IR, and olaparib and PI-103 and IR. Cells were treated with olaparib (1 μM) and PI-103 (0.4 μM) 2 hours before IR, as described in previous studies [18,19]. A specified number of cells were seeded in six-well plates and irradiated with 6 MV x-ray from a linear accelerator (Varian Medical Systems, Palo Alto, CA, USA) at a dose rate of 2.46 Gy/min. After 22 hours incubation, medium was replaced with drug-free, FBS-containing medium, and cells were incubated for 14 to 21 days to allow colony formation. Colonies were fixed in methanol and stained with 0.5% crystal violet, and the number of colonies containing at least 50 cells was determined and the surviving fraction was calculated. Survival data was fitted to a linear-quadratic model using Kaleidagraph version 3.51 (Synergy Software, Reading, PA, USA). Each point on the survival curve represents the mean surviving fraction (SF) from at least three dishes. The sensitizer enhancement ratio 0.05 (SER_0.05_) was defined as the ratio of the isoeffective dose at SF 0.05 in the absence of inhibitors to that in the presence of inhibitors. The average SF relative to the radiation-alone group (SF_O_) at each radiation dose was calculated. Expected SF for the two-drug combination (SF_E_) was calculated as the product of the SF_O_s of the individual single-drug groups. The synergistic index (SI) was calculated as SF_E_/SF_O_, and SI >1.00 indicates a synergistic effect [20].

### Western blotting

Cells were washed, harvested by scraping, and resuspended in lysis buffer (iNtRON Biotechnology, Seongnam, Korea). Proteins were solubilized by sonication, and equal amounts of protein were separated by SDS-PAGE and electroblotted onto polyvinylidenedifluoride membranes (EMD Millipore, Billerica, MA, USA). Membranes were blocked in phosphate-buffered saline (PBS) containing 0.1% Tween 20 and 5%nonfat powdered milk, and probed with primary antibody directed against p-AKT (Ser473), p-ERK (Tyr202/204), Rad51 (all from Cell Signaling Technology, Danvers, MA, USA),BRCA1, p-DNA-protein kinase (PK; Ser2056), PAR (all from Abcam, Cambridge, UK), and β-actin (Santa Cruz Biotechnology, Santa Cruz, CA, USA). Membranes were washed and incubated with secondary antibody consisting of peroxidase-conjugated goat anti-rabbit or anti-mouse IgG(Jackson ImmunoResearch Laboratories, West Grove, PA, USA) at a dilution of1:10,000 for 1 hour. Membrane washing and western blotting was performed using an ECL kit (iNtRON Biotechnology, Seongnam, Korea).

### Total RNA extraction and reverse transcription

Total cellular RNA was isolated using an RNeasy Mini Kit (Qiagen, Carlsbad, CA, USA), and cDNA was made using M-MLV reverse transcriptase (M1705, Promega, Madison, WI, USA) according to manufacturers’ instruction.

### Quantitative real-time polymerase chain reaction (PCR)

Quantitative real-time reverse transcription (RT) PCR was performed using SYBR Premix Ex Taq (Takara Bio Inc., Otsu, Shiga, Japan). The following primer was used: Rad51 (forward 5′-gcataaatgccaacgatgtg-3′, reverse 5′-atgatctctgaccgcctttg-3′). For quantification, all values were normalized to *β*-actin using the ΔΔCt method with data from 3–5 independent experiments. Data were analyzed using a TP800 (Takara Bio Inc., Otsu, Shiga, Japan).

### Immunohistochemical analysis of γH2AX

Cells were cultured and treated on chamber slides. Seventeen hours after irradiation, cover slips were rinsed, and cells were fixed in 4% paraformaldehyde and permeablized in methanol for 20 minutes. Cells were subsequently washed and blocked in PBS containing 2% bovine serum albumin for 1 hour. Primary antibody against γH2AX (Cell Signaling Technology) was applied to the cells and incubated overnight. Secondary AlexaFluor 488-conjugated donkey anti-goat antibody (Molecular Probes, Eugene, OR, USA) was applied and incubated for 1 hour. Nuclei were counterstained with 4′,6-diamidino-2-phenylindole (DAPI) by incubation of cells in 1 μg/mL DAPI for 5 minutes. Slides were examined on a ZeissAxio Scope.A1 Imager fluorescence microscope. Images were captured using AxioCamMRc5 and AxioVision v.4.4 acquisition software (Carl Zeiss, Jena, Germany).

### In vivo tumor model

Animal experimentation was performed according to a protocol approved by the Institutional Animal Care and Use Committee of Seoul National University Bundang Hospital (No. BA1103-078/015-01).

### Cell labeling and implantation

MDA-MB-435S cells were transfected with a pGL4 luciferase reporter vector (Promega, Madison, WI, USA) according to the manufacturer’s protocol. Nude mice were anesthetized and immobilized, and transfected MDA-MB-435S cells were subcutaneously implanted. One week after implantation, intraperitoneal administration of olaparib and PI-103 of 10 mg/kg was initiated and carried out three times weekly for 2 weeks. After drug treatment, mice were irradiated three times weekly with 3 Gy per fraction. Mice were then observed for 2 weeks.

### Bioluminescence imaging (BLI)

BLI was carried out using the IVIS Lumina II BLI system (Caliper, Hopkinton, MA, USA) according to the manufacturer’s protocol. One week after tumor cell implantation, baseline imaging was performed and, 2 weeks after IR, follow-up imaging was carried out. Mice were anesthetized and D-luciferin was injected intraperitoneally. Imaging was carried out 5 minutes after luciferin injection and repeated every few minutes to determine the maximum luminescence intensity in photons/second. After image acquisition, a region of interest (ROI) was circumscribed for each tumor and a corresponding tumor-free ROI was circumscribed to generate a background-corrected bioluminescence flux value. The maximum background-corrected value for that tumor during the 30-minute imaging session was used as the maximum bioluminescent value.

### Statistical analysis

Statistical significance was assessed by Student *t*-test and one way analysis of variance (ANOVA) using SPSS ver. 12.0 (SPSS Inc., Chicago, IL, USA).

## Results

### Combined use of olaparib and PI-103 enhanced radiation-induced death of TNBC cells

**Figure 1:**
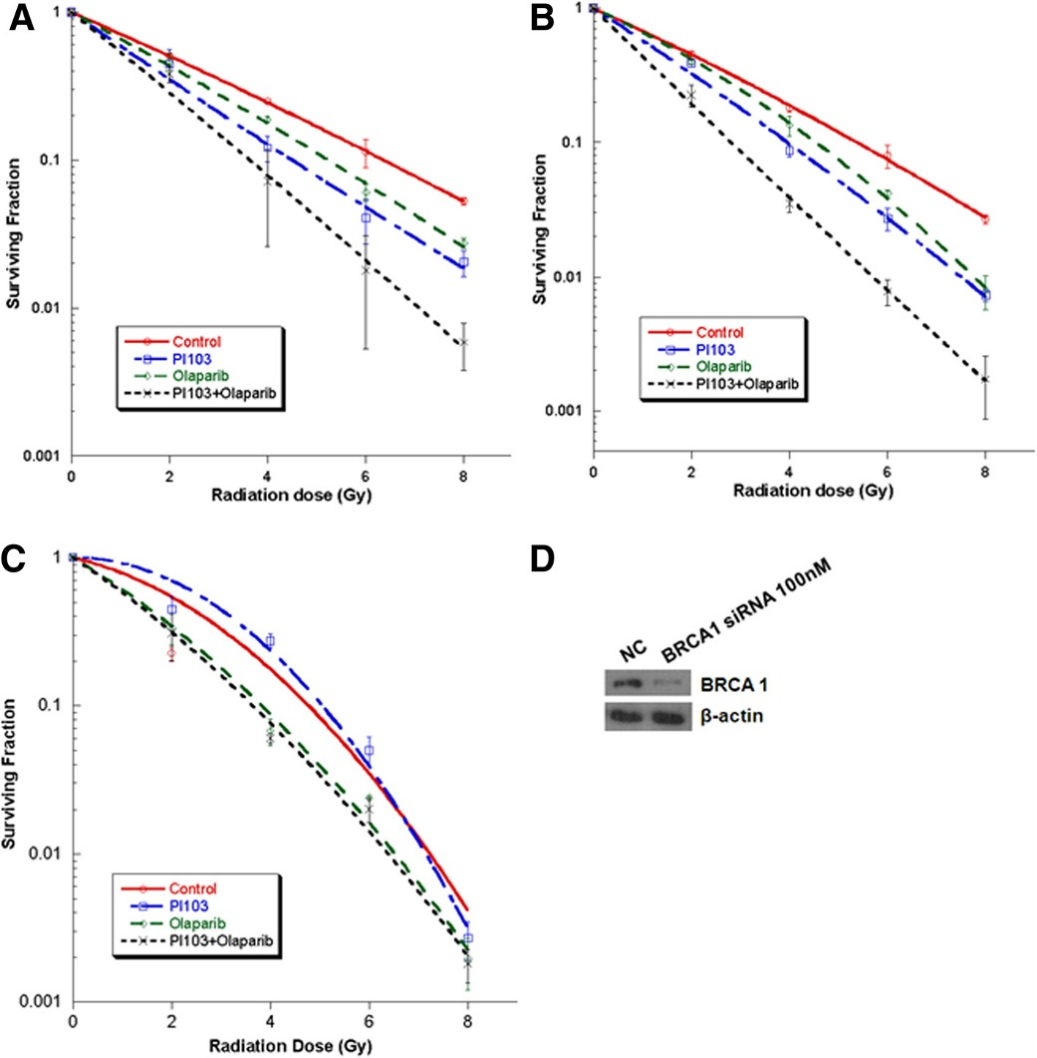
**Effect of olaparib and PI-103 on radiosensitivity of MDA-MB-435S cells.** MDA-MB-435S **(A)**, MDA-MB-231 **(B)**, and MDA-MB-435S cells transfected with BRCA1 siRNA **(C)** were treated with the indicated drugs prior to receiving the indicated dose of radiation. Western blotting showed decreased levels of BRCA1 in cells transfected with BRCA1 siRNA **(D)** compared with untransfected control cells (NC).

To evaluate in vitro radiation sensitization effect of olaparib and PI-103, we performed clonogenic assay. Pretreatment with the combination of olaparib and PI-103 resulted in a significant increase in radiation-induced death of both MDA-MB-435S (SER_0.05_ 1.7) and MDA-MB-231-BR cells (SER_0.05_2.1) (Figure 1A and B). Colony formation in the combined treatment group without radiation was also decreased compared to the control group (*P* = 0.03), but was not significantly different from the single-drug groups (*P* > 0.05). Data was adjusted by control (without radiation) and fitted to a linear quadratic model.

Radiation-induced cell death with 8 Gy was significantly enhanced in the combination treatment group compared to the single-drug groups for both MDA-MB-435S cells (*P* =0.005 for PI-103 and *P* < 0.001 for olaparib) and MDA-MB-231-BR cells (*P* < 0.001 for PI-103 and *P* =0.01 for olaparib) based on *t*-test. The SI of combination treatment at each radiation dose was >1.00 (1.07, 1.27, 1.20, and 1.81 at 2, 4, 6, and 8 Gy, respectively).

To compare these results with results of the same treatments in BRCA-deficient cells, MDA-MB-435S cells were transfected with BRCA1 siRNA. We thought naturally occurring BRCA mutated cells could have many different molecular characteristics other than BRCA. In order to exclude confounding factors we chose siRNA transfection in the same cell lines instead of using naturally occurring BRCA mutated cell lines. As shown in Figure 1C, there appeared to be a radiosensitizing effect with olaparib treatment in the BRCA1-knockdown cells (SER_0.05_ 1.24) compared with the control. However, addition of PI-103 to the olaparib treatment did not result in further enhancement of the radiation-induced cell death.

### Combined use of olaparib and PI-103 enhanced in vivo radiation-induced cell death

**Figure 2:**
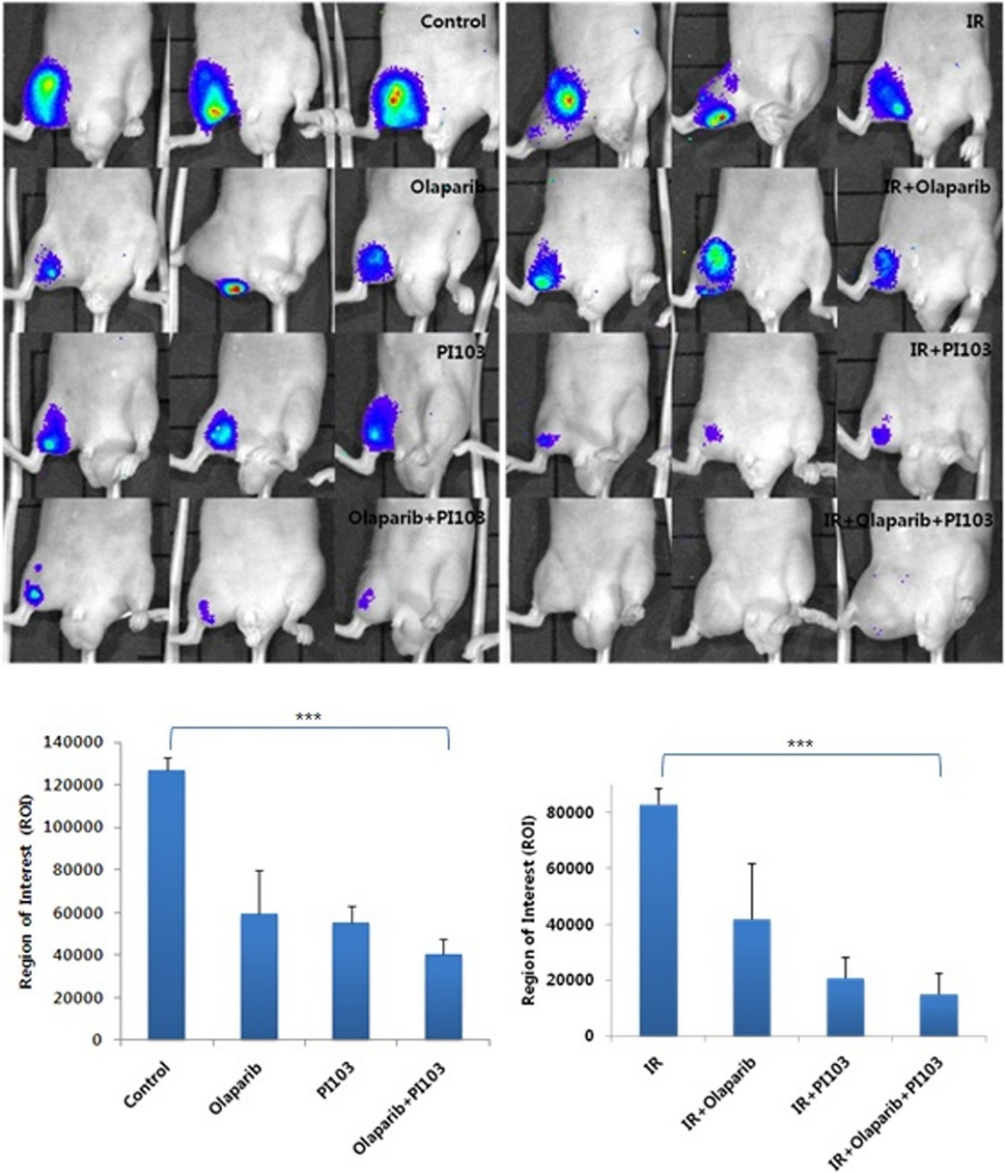
**In vivo radiosensitizing effect of combined use of olaparib and PI-103.** Bioluminescence imaging of nude mice implanted with MDA-MB-435S cells transfected with pGL4 luciferase reporter vector and treated with indicated drugs alone (upper left) or prior to irradiation (IR) (upper right) and quantitation (lower panels). A marked decrease in tumor volume was induced with combined use of olaparib and PI-103, compared to radiation alone (*P* < 0.001 by *t*-test). Means of ROI values were significantly different between groups (*P* < 0.001). Y-axes show bioluminescence in photons/second. *P* < 0.001, ***.

After confirming the in vitro radiosensitizing effect of combination treatment with olaparib and PI-103, we investigated the in vivo effect. MDA-MB-435S cells transfected with a luciferase reporter vector were implanted in nude mice, which were treated with olaparib and PI-103 alone or together, with or without radiation. Two weeks after commencement of the treatment, the size of tumors was examined in vivo using the IVIS Lumina II BLI system. As shown in Figure 2, a marked decrease in tumor volume was induced with combined use of olaparib and PI-103, compared to radiation alone (*P* < 0.001 by *t*-test). Means of ROI values were significantly different between the groups (*P* < 0.001 by one way ANOVA).

### PI-103 induced persistent γH2AX foci

According to previous studies, radiation induces DNA damage and PARP/PI3K inhibition impairs DNA damage repair [14-16]. We detected γH2AX foci to evaluate the capacity of repair of radiation induced DNA damage in each treatment groups.

**Figure 3:**
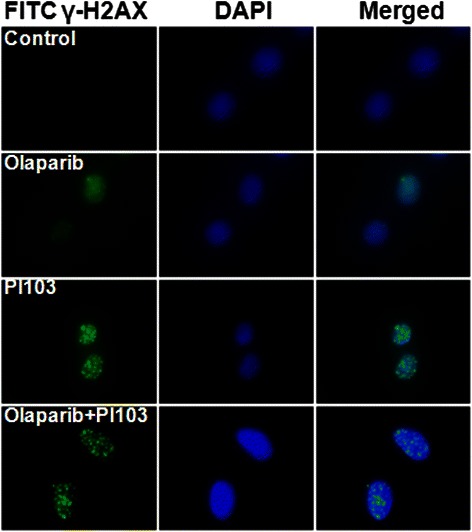
**Immunofluorescence-based detection of γH2AX.** γH2AX was detected in MDA-MB-435S cells treated with the indicated drugs prior to irradiation (IR). Nuclei were counterstained with DAPI.

Pretreatment with PI-103, alone and together with olaparib, followed by irradiation caused marked prolongation of γH2AX foci formation, indicating delayed DNA repair, whereas pretreatment with olaparib alone followed by irradiation showed relatively few foci 17 hours after treatment (Figure 3).

**Figure 4:**
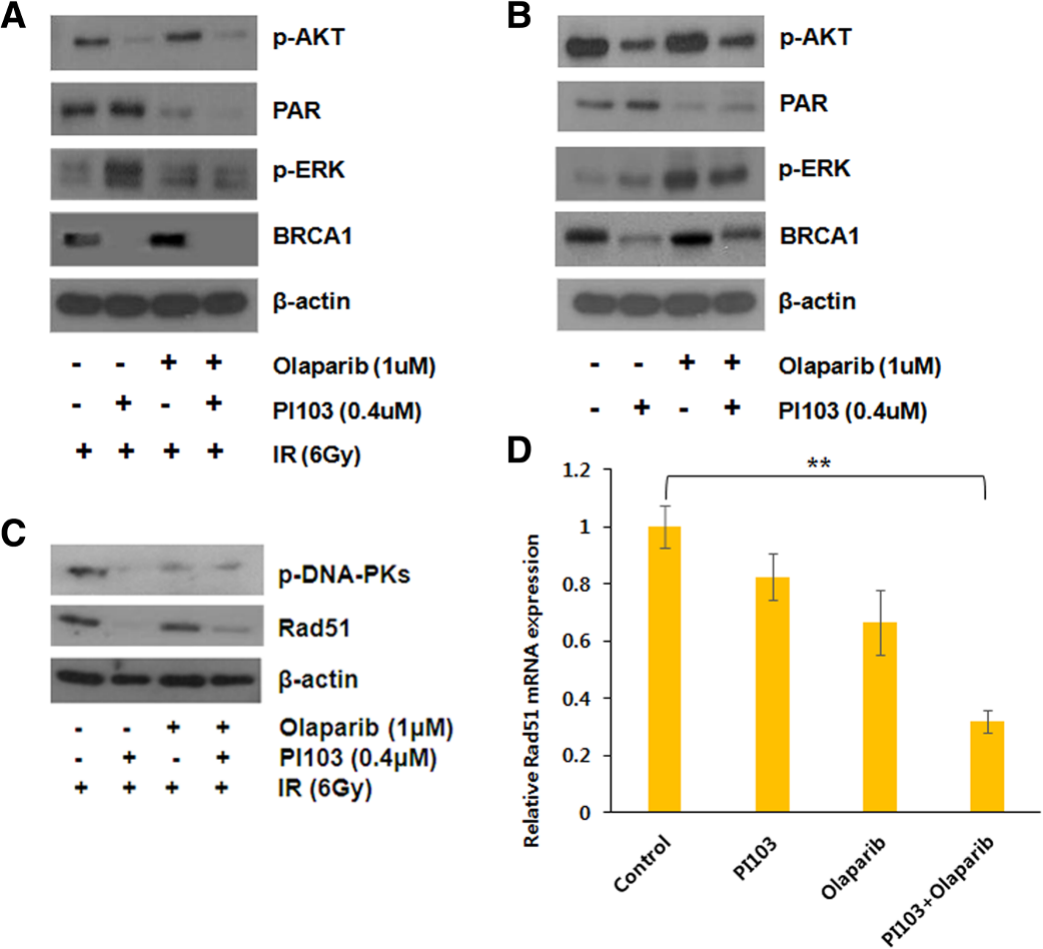
**Western analysis of drug- and radiation-treated cells.** The indicated proteins were detected by western analysis of MDA-MB-435S cells treated with the indicated drugs prior to irradiation (IR) **(A, B, C)**. β-actin served as internal control. Relative Rad51 mRNA expression level was measured by quantitative real-time reverse transcription polymerase chain reaction in MDA-MB-435S cells treated with the indicated drugs **(D)**. *P* < 0.001, **.

Based on the results observed with PI-103 pretreatment, we evaluated the molecules involved in DNA repair. Pretreatment with PI-103 was associated with decreased p-DNA-PK and Rad51 (Figure 4C). Quantitative real-time RT PCR data showed decreased mRNA expression of Rad51 with treatment with PI-103 and olaparib (*p* = 0.001 by *t*-test, Figure 4D).

### PI-103 induced upregulation of ERK and downregulation of BRCA1

Decreased levels of p-AKT and PAR induced by treatment with PI-103 and olaparib, respectively, showed that the drugs were working well (Figure 4A,B). To investigate the possible mechanisms of radiosensitization, we analyzed changes in the candidate proteins with reference to the study of Ibrahim et al. [15]. It is a well-known phenomenon that inhibition of the PI3K signaling pathway induces activation of the ERK pathway [21,22]. As expected, treatment with PI-103 induced elevation of p-ERK level. Pretreatment with PI-103 was associated with activation of ERK and downregulation of BRCA1, whereas the increased level of PAR observed with PI-103 treatment disappeared with the addition of olaparib (Figure 4A,B).

## Discussion

TNBC is known to have aggressive clinical behaviors and high mortality rates [1]. A high incidence and early development of distant metastasis to areas, such as brain or lung, in TNBC or basal-like breast cancer cases have been reported in the literature, and the median duration of survival after distant metastasis was significantly shorter than that of other subtypes [23]. In addition, the basal-like subtype was associated with an increased risk of local and regional relapse after surgery, based on multivariate analysis [24]. Furthermore, lack of specific treatment targets, such as ER or HER2, presents a major problem for the treatment of TNBC patients. To reduce locoregional relapse and to treat brain metastasis or other oligometastasis, the effect of radiation on TNBC needs to be enhanced.

PARP inhibitor has shown a significant clinical benefit in BRCA-related TNBC patients [25]. However, in clinical situations, carriers of BRCA mutation account for only a part of TNBC or basal-like breast cancer patients, so we also need to focus on the treatment of BRCA-proficient TNBC. As BRCA-mutated breast cancer and sporadic basal-like or TNBC share many clinical and pathological similarities [11,12], use of PARP inhibitors was expected to usher in a new era in the treatment of TNBC. However, clinical outcomes of treatment with PARP inhibitor alone were disappointing with sporadic TNBC. To enhance the efficacy, studies on the combined use of PARP inhibitor and other treatments are ongoing.

Herein, we assessed the radiosensitizing effect of combined treatment with olaparib and PI-103in BRCA-proficient TNBC cell lines derived from metastatic sites. Survival curves generated using the clonogenic assay showed increased radiation-induced cell death with the combined treatment of olaparib and PI-103 in both MDA-MB-435S (Figure 1A) and MDA-MB-231-BR (Figure 1B) cells. The MDA-MB-231-BR cell line is a subclone of MDA-MB-231 that selectively metastasizes to the brain, demonstrating that this combined targeting strategy may be applied to enhance the effects of radiation on brain metastasis in TNBC patients.

Therapeutic radiation induces DNA damage with single strand breaks (SSBs) and DSBs. PARP1 detects SSBs, binds to the DNA break sites, and is then auto-poly(ADP-ribosyl)ated and recruits the enzymes required to form the BER multi-protein complex [6,7]. DSBs are repaired by nonhomologous end joining (NHEJ) and HR. NHEJ repairs most radiation-induced DSBs, does not require a template, and may occur during any stage of the cell cycle. In contrast, HR is an error-prone process, requires a sister chromatid as a template, and thus can only occur during the S and G2 phases [16,26].

Under normal conditions, SSBs are efficiently repaired and do not lead to significant cell death. However, unrepaired SSBs or delayed repair of SSBs during the DNA replication process can induce DSBs, which must be repaired by HR. Therefore, PARP inhibition is an effective treatment strategy for tumors with HR deficiency, such as those with a BRCA mutation. As expected, siRNA-mediated knockdown of BRCA1 caused a high degree of radiosensitization in itself, and addition of olaparib further enhanced the effects of radiation, as shown in Figure 1C.

Radiation induces DNA damage, and PARP inhibitor suppresses DNA repair. Thus, radiation and PARP inhibitor would have synergistic effects in killing tumor cells, and many preclinical studies have shown increased radiosensitization with the use of PARP inhibitor on replicating cells [16]. Generally, tumors have a higher proportion of replicating cells than the surrounding normal tissues, making this strategy very attractive in the field of radiation oncology. Consistent with other studies, we also observed in vitro and in vivo radiosensitizing effects of PARP inhibition.

In addition, targeting of the PI3K signaling pathway is a well-known strategy to enhance radiation sensitivity, based on the finding that PI3K controls DNA DSB repair [14]. PI-103 is a potent inhibitor of class I PI3Ks/mTOR/DNA-PK. Pretreatment with PI-103 could impair DNA repair via inhibition of the PI3K signaling pathway and DNA-PK. As expected, PI-103 delayed DNA repair and was associated with decreased RAD51 and p-DNA-PK in the MDA-MB-435S cells tested, as shown in the current study (Figure 4C). Under impaired conditions of DNA DSB repair caused by PI-103, cells may become more dependent on BER. Consequently, pretreatment with PI-103 induced increased PAR levels (i.e., increased PARP activity) in this study (Figure 4), as seen in other studies [15,27]. This increased activation of PARP was completely blocked by adding olaparib, and the combined use of PI-103 and olaparib showed increased effects of radiation treatment in both in vitro and in vivo models.

It is well known that inhibition of the PI3K signaling pathway induces compensatory activation of the ERK pathway [21,22], and results also revealed elevated p-ERK after treatment with PI-103 (Figure 4A). Abnormally high ERK activity was generally thought to be associated with cancer cell survival and progression [28], but the recent study of Ibrahim et al. showed the interesting result that PI3K inhibition impaired BRCA1/2 expression via elevated ERK and sensitized BRCA-proficient TNBC to PARP inhibition [15]. They measured BRCA1/2 mRNA levels in several BRCA-proficient TNBC cell lines (MDA-MB-468, MDA-MB-231, HCC1143, and HCC70) treated with the pan-PI3K inhibitor NVP-BKM120 by quantitative real-time PCR, and decreased BRCA1/2 mRNA level occurred in all of the tested cell lines. Inhibition of the PI3K signaling pathway induces feedback upregulation of ERK and subsequent increased activation of the ERK-related transcription factor ETS1. ETS1 is a negative regulator of BRCA1/2 expression, and elevated ETS1 suppresses BRCA1/2 expression. Consequently, the impaired HR sensitizes the cells to PARP inhibitors. As in Ibrahim’s study, it was also revealed that PI-103 induces downregulation of the PI3K pathway, upregulation of p-ERK, and decreases BRCA1 levels (Figure 4A).Further, adding PI-103 to olaparib did not result in further enhancement of radiation-induced cell death compared to olaparib alone in MDA-MB-435Scells transfected with BRCA1 siRNA (Figure 1C), supporting the hypothesis that PI3K inhibition results in HR impairment via BRCA downregulation.

## Conclusions

In summary, the combined use of olaparib and PI-103 enhanced radiation-induced cell death in BRCA-proficient MDA-MB-435S and MDA-MB-231-BR cell lines and xenografts. TNBC patients have high incidences of locoregional relapse and distant metastasis, and radiation therapy is involved not only in locoregional control, but also in the treatment of distant recurrences, such as brain metastasis or other oligometastasis. Targeting of the PI3K signaling pathway combined with PARP inhibition may be a feasible approach to enhance effects of radiation on BRCA-proficient TNBC.

## Abbreviations

TNBC: Triple negative breast cancer
PARP: Poly(ADP-ribose) polymerase
PI3K: Phosphoinositide 3-kinase
IR: Irradiation
ER: Estrogen receptor
HER2: Human epidermal growth factor 2
BER: Base excision repair
HR: Homologous recombination
DSB: Double-strand break
ERK: Extracellular signal-regulated kinase
SER: Sensitizer enhancement ratio
SF: Surviving fraction
SI: Synergistic index
RT: Reverse transcription
PCR: Polymerase chain reaction
BLI: Bioluminescent imaging
SSB: Single-strand break
NHEJ: Nonhomologous end joining

## References

[1] Dent R, Trudeau M, Pritchard KI, Hanna WM, Kahn HK, Sawka CA (2007). Triple-negative breast cancer: clinical features and patterns of recurrence. Clin Cancer Res.

[2] Hudis CA, Gianni L (2011). Triple-negative breast cancer: an unmet medical need. Oncologist.

[3] Pieper AA, Verma A, Zhang J, Snyder SH (1999). Poly (ADP-ribose) polymerase, nitric oxide and cell death. Trends Pharmacol Sci.

[4] Shall S, de Murcia G (2000). Poly(ADP-ribose) polymerase-1: what have we learned from the deficient mouse model?. Mutat Res.

[5] Ziegler M, Oei SL (2001). A cellular survival switch: poly(ADP-ribosyl)ation stimulates DNA repair and silences transcription. BioEssays.

[6] Dantzer F, Schreiber V, Niedergang C, Trucco C, Flatter E, De La Rubia G (1999). Involvement of poly(ADP-ribose) polymerase in base excision repair. Biochimie.

[7] Fortini P, Pascucci B, Parlanti E, D’Errico M, Simonelli V, Dogliotti E (2003). The base excision repair: mechanisms and its relevance for cancer susceptibility. Biochimie.

[8] Tucker CL, Fields S (2003). Lethal combinations. Nat Genet.

[9] Comen EA, Robson M (2010). Inhibition of poly(ADP)-ribose polymerase as a therapeutic strategy for breast cancer. Oncology.

[10] Gage M, Wattendorf D, Henry LR (2012). Translational advances regarding hereditary breast cancer syndromes. J Surg Oncol.

[11] Anders CK, Winer EP, Ford JM, Dent R, Silver DP, Sledge GW (2010). Poly(ADP-Ribose) polymerase inhibition: “targeted” therapy for triple-negative breast cancer. Clin Cancer Res.

[12] Turner N, Tutt A, Ashworth A (2004). Hallmarks of ‘BRCAness’ in sporadic cancers. Nat Rev Cancer.

[13] Gelmon KA, Hirte HW, Robidoux A, Tonkin KS, Tischkowitz M, Swenerton K (2010). Can we define tumors that will respond to PARP inhibitors- A phase II correlative study of olaparib in advanced serous ovarian cancer and triple-negative breast cancer. J Clin Oncol.

[14] Kumar A, Fernandez-Capetillo O, Carrera AC (2010). Nuclear phosphoinositide 3-kinase beta controls double-strand break DNA repair. Proc Natl Acad Sci U S A.

[15] Ibrahim YH, Garcia-Garcia C, Serra V, He L, Torres-Lockhart K, Prat A (2012). PI3K inhibition impairs BRCA1/2 expression and sensitizes BRCA-proficient triple-negative breast cancer to PARP inhibition. Cancer Discovery.

[16] Chalmers AJ, Lakshman M, Chan N, Bristow RG (2010). Poly(ADP-ribose) polymerase inhibition as a model for synthetic lethality in developing radiation oncology targets. Semin Radiat Oncol.

[17] Kim IA, Shin JH, Kim IH, Kim JH, Kim JS, Wu HG (2006). Histone deacetylase inhibitor-mediated radiosensitization of human cancer cells: class differences and the potential influence of p53. Clin Cancer Res.

[18] Choi EJ, Ryu YK, Kim SY, Wu HG, Kim JS, Kim IH (2010). Targeting epidermal growth factor receptor-associated signaling pathways in non-small cell lung cancer cells: implication in radiation response. Mol Cancer Res.

[19] Senra JM, Telfer BA, Cherry KE, McCrudden CM, Hirst DG, O’Connor MJ (2011). Inhibition of PARP-1 by olaparib (AZD2281) increases the radiosensitivity of a lung tumor xenograft. Mol Cancer Ther.

[20] Elias L, Crissman HA (1988). Interferon effects upon the adenocarcinoma 38 and HL-60 cell lines: antiproliferative responses and synergistic interactions with halogenated pyrimidine antimetabolites. Cancer Res.

[21] Chandarlapaty S, Sawai A, Scaltriti M, Rodrik-Outmezguine V, Grbovic-Huezo O, Serra V (2011). AKT inhibition relieves feedback suppression of receptor tyrosine kinase expression and activity. Cancer Cell.

[22] Serra V, Scaltriti M, Prudkin L, Eichhorn PJ, Ibrahim YH, Chandarlapaty S (2011). PI3K inhibition results in enhanced HER signaling and acquired ERK dependency in HER2-overexpressing breast cancer. Oncogene.

[23] Kennecke H, Yerushalmi R, Woods R, Cheang MC, Voduc D, Speers CH (2010). Metastatic behavior of breast cancer subtypes. J Clin Oncol.

[24] Voduc KD, Cheang MC, Tyldesley S, Gelmon K, Nielsen TO, Kennecke H (2010). Breast cancer subtypes and the risk of local and regional relapse. J Clin Oncol.

[25] Tutt A, Robson M, Garber JE, Domchek SM, Audeh MW, Weitzel JN (2010). Oral poly(ADP-ribose) polymerase inhibitor olaparib in patients with BRCA1 or BRCA2 mutations and advanced breast cancer: a proof-of-concept trial. Lancet.

[26] Jackson SP (2002). Sensing and repairing DNA double-strand breaks. Carcinogenesis.

[27] Juvekar A, Burga LN, Hu H, Lunsford EP, Ibrahim YH, Balmana J (2012). Combining a PI3K inhibitor with a PARP inhibitor provides an effective therapy for BRCA1-related breast cancer. Cancer Discovery.

[28] Dhillon AS, Hagan S, Rath O, Kolch W (2007). MAP kinase signalling pathways in cancer. Oncogene.

